# Hereditary angioedema (HAE) in children and adolescents—a consensus on therapeutic strategies

**DOI:** 10.1007/s00431-012-1726-4

**Published:** 2012-04-29

**Authors:** V. Wahn, W. Aberer, W. Eberl, M. Faßhauer, T. Kühne, K. Kurnik, M. Magerl, D. Meyer-Olson, I. Martinez-Saguer, P. Späth, P. Staubach-Renz, W. Kreuz

**Affiliations:** 1 Department of Pediatric Pneumology and Immunology, Campus Virchow Hospital, Charité Medical University, Augustenburger Platz 1, 13353 Berlin, Germany; 2 Department of Dermatology and Venereology, Medical University of Graz, Graz, Austria; 3 Department of Pediatrics, City Hospital, Braunschweig, Germany; 4 Division of Pediatrics, Department of Pediatric Rheumatology, Immunology and Infectiology, Municipal Hospital “St. Georg”, Leipzig, Germany; 5 University Children’s Hospital, Basel, Switzerland; 6 Dr. von Haunersches Children’s Hospital, Inner City Medical Center of the Ludwig Maximilian University, Munich, Germany; 7 Allergy Center, Charité Medical University, Berlin, Germany; 8 Department of Immunology and Rheumatology, Hannover Medical School, Hannover, Germany; 9 Johann Wolfgang Goethe University Hospital, Frankfurt am Main, Germany; 10 Institute of Pharmacology, University of Bern, Bern, Switzerland; 11 Department of Dermatology, University Hospital Mainz, Mainz, Germany

**Keywords:** C1-INH (C1 inhibitor, C1-esterase inhibitor), Hereditary angioedema, Consensus, Pediatric

## Abstract

Hereditary angioedema due to C1 inhibitor (C1 esterase inhibitor) deficiency (types I and II HAE-C1-INH) is a rare disease that usually presents during childhood or adolescence with intermittent episodes of potentially life-threatening angioedema. Diagnosis as early as possible is important to avoid ineffective therapies and to properly treat swelling attacks. At a consensus meeting in June 2011, pediatricians and dermatologists from Germany, Austria, and Switzerland reviewed the currently available literature, including published international consensus recommendations for HAE therapy across all age groups. Published recommendations cannot be unconditionally adopted for pediatric patients in German-speaking countries given the current approval status of HAE drugs. This article provides an overview and discusses drugs available for HAE therapy, their approval status, and study results obtained in adult and pediatric patients. Recommendations for developing appropriate treatment strategies in the management of HAE in pediatric patients in German-speaking countries are provided.*Conclusion* Currently, plasma-derived C1 inhibitor concentrate is considered the best available option for the treatment of acute HAE-C1-INH attacks in pediatric patients in German-speaking countries, as well as for short-term and long-term prophylaxis.

## Introduction

Hereditary angioedema (HAE) is a rare disease that usually manifests in childhood or adolescence. The pathogenesis is either related to insufficient levels of functional C1 inhibitor (C1-INH) (type I HAE) or normal levels of dysfunctional C1-INH (type II HAE). Patients with type III HAE are mainly women and have normal levels of functional C1-INH, although some will demonstrate activating mutations in the coagulation factor XII (Hageman factor; HAE-FXII). Type III is essentially irrelevant in children and adolescents. For that reason, this consensus will focus solely on types I and II HAE.

The characteristic symptoms of HAE include recurrent spontaneous swellings of the skin and mucosal tissues that mimic allergic Quincke edema. However, in contrast to allergic reactions, these swelling sites tend to be pale, hard, and doughy, sometimes causing considerable tension-induced pain. The fact that they do not cause itching may be helpful in an emergency setting to aid the differential diagnosis in contrast to an allergic reaction. Urticaria is atypical for C1-INH deficiency, while erythema marginatum-like erythema has been observed in individual patients prior to the onset of an attack. Without knowledge of the underlying disease, abdominal colic caused by swelling attacks in the bowel may give rise to surgical intervention (mostly for appendicitis or mechanical ileus). An abdominal ultrasound can provide clues as to whether the typical cocarde reaction occurs in the appendix or whether HAE-related swelling has caused thickening of the bowel wall. Swellings in the genital region may also occur, and laryngeal swelling can be life-threatening. Deaths by asphyxiation following laryngeal swelling have been reported in many families, including a 9 year-old boy who died from a laryngeal swelling that was the first clinical sign of his C1-INH deficiency [1–4]. If left untreated, attacks usually occur intermittently and are subject to great interindividual variability [33]. Classically, the attacks develop slowly over 24 h and resolve spontaneously without intervention after another 2 to 3 days.

The trigger of the attacks cannot always be determined. Minor injury, dental work, an infection (e.g., Epstein–Barr virus or *Helicobacter pylori*), mental stress, or drugs like angiotensin-converting enzyme inhibitors can all precipitate an attack. Most contraceptives (apparently not progestin-only products with pure progestin) and menstruation have been occasionally identified as triggers.

Unfortunately, it often takes many years until the diagnosis of HAE is established. Yet, the earliest possible diagnosis can help avoid administration of ineffective therapies and, in isolated cases, even prevent life-threatening situations such as laryngeal edema. When an HAE patient has been identified in a family, it is imperative that the entire family, including any infants and toddlers, be examined. Indeed, there is often an autosomal-dominant pattern of inheritance (see the Online Mendelian Inheritance in Man database, entry 106100). If the disease can be diagnosed successfully before patients experience their first severe swelling attack, effective preventive or therapeutic measures can be taken.

In 2010, consensus recommendations for therapeutic strategies were issued for all age groups [6] and a pediatric consensus was published in 2005 [7]. In spite of this, the group of experts (pediatricians and dermatologists) who met in Berlin on 23 June 2011 to discuss a therapeutic strategy for pediatric patients that is compatible with the marketing approval status in these countries were concerned whether the existing international consensus papers can be unconditionally adopted for children and adolescents in German-speaking countries due to both medical and legal issues. The aims of the consensus meeting were:

1. to present all drugs available for the therapy of HAE and discuss the study results obtained in adults and children along with the adverse effects of these forms of therapy.
2. to determine which of these available drugs are approved in German-speaking countries and integrate them into a therapeutic strategy for managing HAE in children and adolescents.
3. to brainstorm how to further improve HAE therapy in children and adolescents in the future.

The experts at the meeting also intentionally addressed compounds that are presently approved for adults only because pediatricians should be able to answer questions concerning the therapeutic options available to their patients post-adolescence. Moreover, it is anticipated that further approvals will be granted for children and adolescents as soon as the corresponding study results become available.

Experts participating in the consensus meeting unanimously agreed that, *under all circumstances, the current drug approvals in the individual countries should be considered* before deciding, in isolated cases, on off-label administration of therapeutics that are only approved in other countries.

## Approval status and overview of HAE-C1-INH therapeutics—current data from clinical studies in children and adults, including risks

Table 1 presents an overview of the current approval status of drugs used in the management of HAE. The individual HAE therapeutics and study results obtained in adults and children are summarized below; an overview of specific risks and adverse events for the treatment with the available drugs is provided in Table 2 and an assessment on use in pediatrics is provided in Table 3.

**Table 1:** Approval status of products for the treatment of HAE-C1-INH (Europe and USA) < 18 years of age

Active ingredient/trade name	Approval in	Approval for pediatric patients	Indication in HAE-C1-INH	Route of administration
Human pdC1-INH concentrates				
Berinert®	Europe, USA	Children and adolescents	Acute attack, home therapy^a^	Intravenous
Cinryze®	Europe^b^	Adolescents	Acute attack, short-term and long-term prophylaxis, home therapy	Intravenous
	USA	Adolescents	Long-term prophylaxis	Intravenous
Recombinant human C1-INH concentrate				
Ruconest®	Europe	No	Acute attack	Intravenous
Kallikrein inhibitor and bradykinin receptor antagonists				
Icatibant/Firazyr®	Europe, USA	No	Acute attack, home therapy	Subcutaneous
Ecallantide/Kalbitor™	USA	Adolescents >16 years of age	Acute attack	Subcutaneous
Attenuated androgens^c^				
Danazol/Danatrol®	Switzerland	Adolescents	Long-term prophylaxis	Oral
Danazol/Danokrin®	Austria	No	Long-term prophylaxis	Oral
Danazol/Danocrine™	USA	No specific approval for children and adolescents	Long-term prophylaxis^d^	Oral
Stanozolol/Winstrol™	USA	Children and adolescents	Long-term prophylaxis	Oral
Antifibrinolytics^e^				
Tranexamic acid/Cyklokapron®	Austria	No specific approval for children and adolescents	HAE	Oral
	Switzerland	Children and adolescents	Long-term prophylaxis, acute attacks (for prodromal symptoms)	Oral
	Germany	Children and adolescents	Long-term prophylaxis, short-term prophylaxis possible	Oral

^a^Home therapy is approved in 23 European countries (but not in Switzerland)

^b^Not yet approved in Switzerland

^c^Not approved in Germany

^d^Recommendation to elevate doses for breakthrough attacks

^e^ε-Aminocaproic acid (Amicar™) is not approved for HAE therapy

**Table 2:** Specific risks and adverse events of products for the treatment of HAE-C1-INH

Active ingredient/trade name	Risks and adverse events
Human pdC1-INH concentrates	
Berinert®	A theoretical risk of pathogen transmission is associated with all plasma products. No such transmissions have thus far been described. In this respect, the product can be judged to be safe. It was speculated that long-term use of Berinert may be associated with an increased frequency of HAE-C1-INH attacks [2].
Cinryze®	A theoretical risk of pathogen transmission is associated with all plasma products. No such transmissions have thus far been described. In this respect, the product can be judged to be safe.
Recombinant human C1-INH concentrate	
Ruconest®	Several patients developed antibodies to rabbit antigens (from dander and hair, not from the C1-INH); allergic reactions were observed rarely.
Kallikrein and bradykinin receptor antagonists	
Icatibant/Firazyr®	From the theoretical perspective, caution is advised in patients with ischemic heart disease, unstable angina pectoris, and in the first weeks following a stroke. Clinically relevant problems in this regard have not been observed to date.
Ecallantide/Kalbitor™	Worth mentioning is the risk of anaphylactic reactions (frequency according to the boxed warning in the USA full prescribing information, 3.9 % [21]).
Attenuated androgens	
Danazol/Danatrol®	The most common adverse effects are virilization, weight gain, menstrual irregularities, depression/aggression, myalgia, and acne. Adverse effects such as hypercholesterolemia, hypertension, erythrocytosis, and hepatic tumors mandate the need for regular medical checkups. Furthermore, in children, growth disorders and premature closure of the epiphyseal cartilage are conceivable and have not been sufficiently analyzed in clinical studies.
Danazol/Danokrin®	
Danazol/Danocrine™	
Stanozolol/Winstrol™	
Antifibrinolytics	
Tranexamic acid/Cyklokapron®	The most common adverse effects are dose-dependent gastrointestinal symptoms (nausea, vomiting, and diarrhea).

**Table 3:** Assessment on pediatric use for the treatment of HAE-C1-INH

Active ingredient/trade name	Assessment on pediatric use
Human pdC1-INH concentrates	
Berinert®	Pediatric data for Berinert indicate that its efficacy and safety of 20 U/kg in pediatric patients are comparable with that for adults.
Cinryze®	Pediatric data for Cinryze indicate that its efficacy, safety, and tolerability in pediatric patients are comparable with that for adults. The recommended dose (1,000 U) is the same for all types of attacks and for all body weights. The paucity of data on body weight-based dosing, as is otherwise common in pediatrics, is seen as critical. No adverse effects in adults and adolescents from high doses have become known to date. Cinryze is not yet approved for the management of acute attacks and for prophylaxis in children.
Recombinant human C1-INH concentrate	
Ruconest®	Until data on children and adolescents are published and approval of the drug is granted, Ruconest should only be used when specifically warranted.
Kallikrein inhibitor and bradykinin receptor antagonists	
Icatibant/Firazyr®	On a preliminary basis, no recommendation favoring use in children and adolescents can be made, given the lack studies in pediatric patients.
Ecallantide/Kalbitor™	The currently available data on the treatment of pediatric patients is insufficient. Moreover, no European approval is available.
Attenuated androgens	
Danazol/Danatrol®	Given their adverse reaction profile, at present, androgens should not be used for the long-term prophylaxis of pediatric HAE-C1-INH. Neither can a recommendation be given for short-term prophylaxis, despite the fact that danazol is approved in Switzerland for children >12 years of age.
Danazol/Danokrin®	
Danazol/Danocrine™	
Stanozolol/Winstrol™	
Antifibrinolytics	
Tranexamic acid/Cyklokapron®	The use of ε-aminocaproic acid cannot be recommended, as it is approved in some countries by regulatory authorities for the treatment of HAE. Due to the doubtful efficacy of tranexamic acid, it should be avoided for short-term and long-term prophylaxis in pediatric patients.

The consensus meeting relating to children and adolescents did not consider data from studies in pregnant and nursing mothers.

### Human plasma-derived C1-INH (pdC1-INH) concentrates

**Fig. 1:**
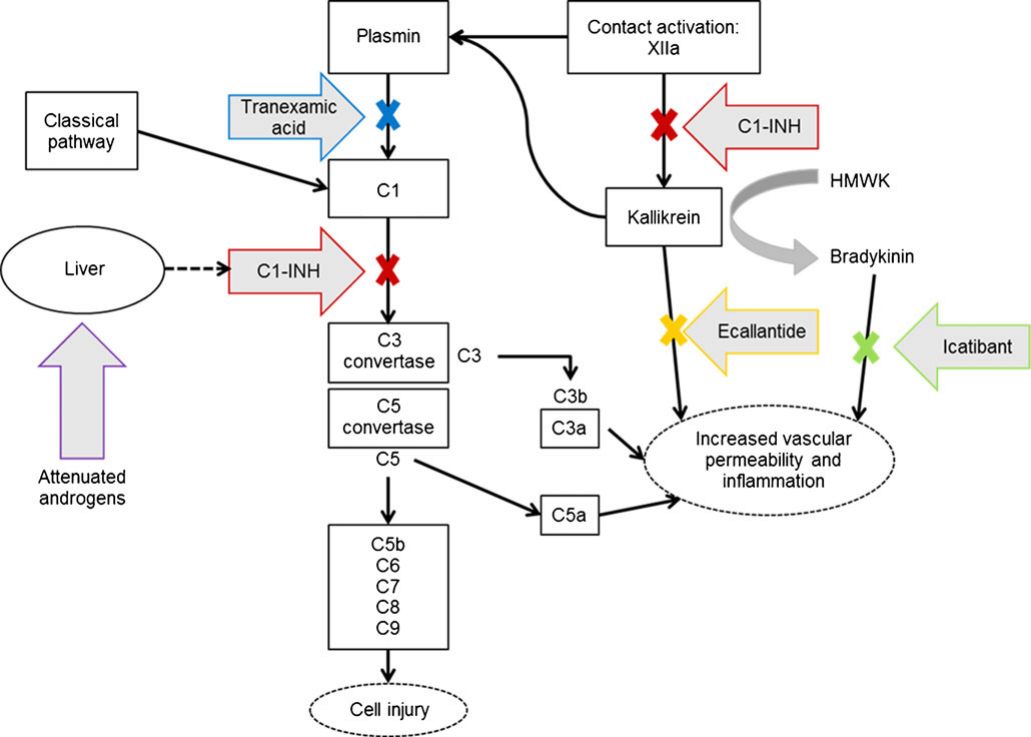
Mechanisms of action for therapeutic agents in treating or preventing HAE. Multiple pathways are capable of complement activation and generating inflammatory mediators including complement anaphylatoxins C3a and, more important, C5a. Activation of the final complement cascade produces a membrane attack complex that produces cellular injury. Angioedema occur after tissue injury from multiple causes. Tissue injury can activate contact activation (Hageman factor or factor XII) to generate kallikrein from prekallikrein, its precursor. Kallikrein in turn generates and activates plasmin from plasminogen, and plasmin can directly activate the C1 esterase complex to initiate complement activation. Under normal circumstances, C1-INH functions to inhibit both complement activation and, to a lesser extent, modulate contact activation. In HAE-C1-INH, because of quantitative or qualitative detective C1-INH, the pathway proceeds unchecked, generating mediators that increase capillary permeability to produce angioedema. Therapeutic approaches are directed at acutely restoring C1-INH levels, inhibiting kallikrein with ecallantide, inhibiting bradykinin with icatibant, and inhibiting plasmin with the lysine analogue tranexamic acid. Attenuated androgens have been used to increase liver synthesis of C1-INH. Figure and description have been adapted from [23]

Human C1-INH, a single-chain glycoprotein consisting of 478 amino acids with a molecular weight of approximately 105 kDa, is a member of the serine protease inhibitor family (SERPIN). Its main function is to inhibit the early activation steps of the complement and kallikrein–kinin systems (see Fig. 1).

#### Berinert®

Berinert is derived from human plasma. The risk of virus transmission is reduced by pasteurization, hydrophobic interaction chromatography, and virus filtration (nanofiltration) using two filters in series. The dosing recommendation is 20 U/kg body weight, intravenous. Berinert is approved for treating acute attacks of HAE in all pediatric age groups (Table 1).

In the randomized, double-blind, placebo-controlled study I.M.P.A.C.T.1, 124 patients (6 to 72 years of age) were treated for each attack (abdominal or facial) with 10 or 20 U/kg of Berinert or with placebo [12]. With 20 U/kg Berinert, the median time to the onset of symptom relief was significantly shorter compared to placebo (0.50 vs. 1.5 h; *p* = 0.0025), whereas this time was not significantly shorter compared to placebo in patients receiving 10 U/kg Berinert (1.2 h). With 20 U/kg Berinert, the median time to the complete resolution of all HAE symptoms was significantly shorter compared to placebo (4.92 vs. 7.79 h; *p* = 0.0237).

In I.M.P.A.C.T.2, an open-label extension study of I.M.P.A.C.T.1, which investigated long-term therapy with Berinert, 1,085 attacks at any body location in 57 patients (10 to 53 years of age) were treated with 20 U/kg of Berinert [13]. The median time to the onset of symptom relief was 0.46 h and was comparable for attacks at all body locations. The median time to complete resolution of all symptoms was 15.5 h (shortest for laryngeal attacks, 5.8 h). In 99 % of all attacks (1,073 of 1,085), a single dose of 20 U/kg was sufficient. One 10 year-old child and six adolescents between 12 and 16 years of age participated in the study; subgroup analyses indicated that the results were comparable across all age groups. There were no serious adverse effects, and no neutralizing anti-C1-INH antibodies were detected.

A retrospective study showed that 500 U of Berinert were efficacious for short-term prophylaxis [16]. In a study on short-term prophylaxis with Berinert in adults and children, swelling attacks occurred in approximately 20 % of 577 tooth extractions performed without short-term prophylaxis, whereas they occurred in <12 % of the 128 tooth extractions with short-term prophylaxis [3]. Evidence for effective long-term prophylaxis with Berinert has been recently published [14, 31].

In a recently published retrospective study, home therapy with Berinert was shown to be effective and safe in children and adolescents [22]. In a pharmacokinetic study in 6 children and 34 adults (receiving on-demand treatment or individual replacement therapy), the median half-life of C1-INH activity was 32.9 h in children and 39.1 h (on-demand therapy) and 30.9 h (individual replacement therapy), respectively, in adult patients [26].

#### Cinryze®

Cinryze is derived from human plasma. The risk of virus transmission is reduced by polyethylene glycol precipitation, pasteurization, and virus filtration (nanofiltration) using two filters in series. The dosing recommendation is 1,000 U (two vials), intravenous, irrespective of the body weight. Cinryze is also approved in adolescents for treating acute attacks of HAE, as well as for short-term and long-term prophylaxis (Table 1).

Two double-blind, randomized, placebo-controlled studies were conducted, one on the treatment of acute swelling attacks and another one on long-term prophylaxis [34]. In both studies, there were no serious adverse effects.

In the acute-treatment study, 71 patients with swelling attacks involving the face, abdomen, or external genitalia were treated with 1,000 U of Cinryze or placebo. Patients not responding within 1 h received a second dose. If the symptoms did not subside or worsened after 4 h elapsed, the patients received another dose of 1,000 U (open-label rescue medication). The median time to onset of unequivocal relief with Cinryze was 2 h (placebo, 4 h; *p* = 0.048). The median time to the complete resolution of symptoms with Cinryze was 12.3 h (placebo, 25.0 h; *p* = 0.004).

In the long-term prophylaxis study, 24 patients (from the acute-treatment study) with at least 2 attacks per month received 1,000 U of Cinryze or placebo every 3 to 4 days (crossover after 12 weeks). During the 12 week treatment period, the number of swelling attacks was significantly lower with Cinryze compared to placebo (mean, 6.1 vs. 12.7 attacks; *p* < 0.0001).

In an interim analysis of an open-label extension study (median duration, 11 months), 88 patients had received Cinryze, some repeatedly, for the treatment of acute attacks, including laryngeal attacks [34]. The repeated treatment also proved effective. Data on home therapy with Cinryze have not been published yet.

In various studies, a total of 46 patients <18 years of age were treated with Cinryze (2 to 5 years, 3 patients; 6 to 11 years, 17 patients; 12 to 17 years, 26 patients) [11]. Overall, the safety, tolerability, and efficacy of Cinryze were comparable with those of adults in both acute treatment and long-term prophylaxis.

### Recombinant human C1-INH concentrate (Ruconest®)

The active ingredient of Ruconest, conestat alfa, is a recombinant analogue of human C1-INH (a single-chain glycoprotein with a molecular weight of 71 kDa). Its glycosylation is not identical to that of human C1-INH, which is presumably the reason for its shorter half-life [17]. Ruconest is produced recombinantly in transgenic New Zealand rabbits and purified from their milk. The dosing recommendation is 50 U/kg, intravenous (maximum dose of 4,200 U in patients with body weight >84 kg, maximum of two doses within of 24 h).

Two randomized, double-blind, placebo-controlled studies have been conducted for dose finding (100 U/kg vs. placebo and 50 vs. 100 U/kg vs. placebo) [35]. Relief of symptoms was similar and significantly faster with 50 and 100 U/kg compared with placebo. There were no serious adverse effects. To date, no data have been published on use in children.

### Kallikrein inhibitor and bradykinin receptor antagonists

#### Icatibant (Firazyr®)

Icatibant, a chemically synthesized decapeptide with a bradykinin-like structure, is a selective antagonist of the bradykinin B2 receptor. Its amino acid sequence makes icatibant resistant to enzymatic degradation. The dosing recommendation is 30 mg, subcutaneous (prefilled syringe), with a maximum of three doses within 24 h.

In the randomized, double-blind studies FAST-1 (placebo-controlled) and FAST-2 (tranexamic acid-controlled), 56 and 74 adults, respectively, received treatment with icatibant or the respective comparator for 1 attack [8]. In FAST-1, the median time to clinically significant improvement was achieved after 2.5 h with icatibant (placebo, 4.6 h) and, in FAST-2, after 2.0 h (tranexamic acid, 12.0 h). No serious adverse effects were observed. A study in children and adolescents is currently in the concrete planning stage.

#### Ecallantide (Kalbitor™)

Ecallantide (DX-88) is a 60-amino-acid recombinant protein that reversibly inhibits the activity of kallikrein [19] (see Fig. 1). The dosing recommendation is 3 × 10 mg given subcutaneously, with a maximum of two doses within 24 h.

Two randomized, double-blind, placebo-controlled studies for approval in the USA with a similar study design have been published on the acute treatment of HAE. In the EDEMA3 study, 71 patients (minimum age, 10 years) were treated with 30 mg ecallantide or placebo for acute attacks [10]. There was a significant improvement in the primary endpoint of the study (treatment outcome score 4 h after administration) for the active drug group compared to placebo.

In the EDEMA4 study in 96 patients, the mean symptom complex severity score and the treatment outcome score showed a significant improvement after 4 h in the active drug group compared to placebo [24]. In a combined analysis of data from EDEMA3 and EDEMA4, the product was also demonstrated to have good efficacy and good tolerability [30]. The most frequent adverse effects were headache, nausea, fever, diarrhea, nasopharyngitis, or local injection-site reactions [10, 19, 24, 30].

### Attenuated androgens

In many parts of the world, androgen derivatives (methyltestosterone, danazol, stanozolol, and oxandrolone) are used for short-term and long-term prophylaxis. C1-INH plasma levels rise with the administration of attenuated androgens, which provides at least a partial explanation for their effect.

The approval status differs in German-speaking countries (see Table 1). For long-term prophylaxis in adolescents, danazol is only approved in Switzerland [28]. The prescribing information of this country states that adolescents >12 years of age should receive an initial daily dose of 400 to 600 mg and that the dose should be incrementally reduced down to the lowest effective dose. Experts explicitly do not recommend androgen therapy for long-term prophylaxis in children [6, 9, 27]. The dose recommended for short-term prophylaxis is 2.5 to 10 mg/kg daily (maximum, 600 mg daily) for 5 days before to 2 days after the intervention [6]. The experts at the current consensus meeting considered this international recommendation as problematic for pediatric patients (see below).

There are no clinical studies in children and adolescents with HAE. A small case collection provides some data on the efficacy and tolerability at low doses [15].

### Antifibrinolytics

Antifibrinolytics (ε-aminocaproic acid or tranexamic acid [cyclic analogue of ε-aminocaproic acid]) are chemically synthesized and exert their action in HAE by inhibiting the conversion of plasminogen to plasmin (see Fig. 1). Antifibrinolytics are less effective than attenuated androgens [6]. The dosing recommendation for tranexamic acid is 20 to 50 mg/kg daily; the lowest effective dose should be aimed for [6].

Earlier double-blind, placebo-controlled studies on these compounds (in 5 to 18 patients, only a few children) showed a reduction in the frequency of attacks [18, 20, 29]. A recent prospective study comparing long-term prophylaxis in patients with and without tranexamic acid did not demonstrate any effect [32].

## Evaluation of recent consensus documents focusing on the differences between children and adults

Two international consensus papers [6, 25] have issued the recommendations summarized below for the treatment of acute attacks, short-term prophylaxis, and long-term prophylaxis.

### Acute attacks

Human pdC1-INH is recommended for all attacks (for adults and children). “Wait and see” is only an option for attacks not involving the face, neck, or abdomen and intestine. Home therapy is recommended for children with frequent and debilitating attacks, under the condition of medically supervised training.

For recombinant C1-INH concentrate (Ruconest), these consensus papers do not give any recommendation whatsoever because this drug first became available in Europe for the treatment of acute attacks in adults in 2010. In an updated version of the consensus paper from 2010, Bowen added recombinant C1-INH concentrate (50 U/kg) for the treatment of acute attacks [5]. To date, there is no experience on its use in children.

### Short-term prophylaxis

In children, pdC1-INH is recommended for short-term prophylaxis. Should pdC1-INH not be available, danazol is also recommended for children. The indication for short-term prophylaxis in children is primarily for surgeries performed in the head and neck area.

### Long-term prophylaxis

The only treatment option for long-term prophylaxis in children is pdC1-INH. Attenuated androgens are not recommended. Tranexamic acid (less effective than attenuated androgens) is recommended for long-term prophylaxis in patients for whom pdC1-INH is not available or in whom attenuated androgens are deemed unacceptable.

At the current pediatric consensus meeting for German-speaking countries, several concerns were raised about these international consensus recommendations:

- Experts strongly advocated taking body weight into account in treatment in pediatrics. Discomfort emerged as to whether appropriate dosing is possible for patients between 12 and 18 years of age with fixed doses established in adults.
- For attenuated androgens, sufficient data are lacking on the long-term safety over decades of use and, additionally, on the growth rate and bone maturation during the growth phase. Should there be a reason for its use in isolated cases, the initial dose (see above) should only be administered for a short period (e.g., for short-term prophylaxis prior to elective procedures). Since approval for children is lacking for long-term use, the issue of dosing will not be addressed further.
- The data available on the efficacy of tranexamic acid are considered insufficient, and therefore, its use cannot be recommended on principle.

## Conclusion

Taking into account the approval status (Table 1) and the published data, the participants at the consensus meeting reached the conclusions summarized in Table 4 regarding recommendations for products for use in children and adolescents.

**Table 4:** Consensus on the treatment of children and adolescents with types I and II HAE-C1-INH

	Therapy for acute attacks			Short-term and long-term prophylaxis
	Peripheral and urogenital	Abdominal	Facial and laryngeal	
“Wait and see”	+/−	+/−	−	n/a
pdC1-INH concentrate	+	+	+	+^a^
Recombinant C1-INH concentrate, Icatibant and Ecallantide^b,c^	−	−	−	−
Attenuated androgens^d^	−	−	−	−
Tranexamic acid	−	−	−	−

n/a not applicable, +/− depending on the intensity

^a^Of the two available products, only Cinryze is approved for long-term prophylaxis

^b^Recombinant C1-INH concentrate, ecallantide, and icatibant are currently not approved for children and adolescents in Europe. Studies in children and adolescents are required

^c^No approval for ecallantide in Europe

^d^Attenuated androgens are not approved in Germany, in Austria not for children

In some situations, a “wait and see” strategy may be utilized, depending on the location and intensity of the attack. This option may be considered if the swelling is not too painful and does not impair the patient’s everyday activities and must be mutually agreed upon between the treating physician and the patient. Attacks that are painful or cause any impairment to the patient’s everyday activities should always be treated.

With all products indicated for the treatment of acute attacks, a further dose should be given whenever the patient fails to noticeably respond to the treatment within 2 to 3 h (i.e., if the swelling continues to get worse).

Home therapy with pdC1-INH is possible for accordingly instructed and educated patients (or their parents or guardians). A home therapy regimen is particularly reasonable when long-term prophylaxis is necessary; although the frequency of attacks (one per week?, two per week?) appropriate for this therapeutic option still needs to be defined. A formal consensus on this was not issued, seeing as this indication is very rare in childhood, because the overwhelming majority of pediatric patients have markedly fewer attacks. While not an evidence-based guideline, the consensus that was reached nevertheless fosters clear-cut decisions for treatment strategies.

## Abbreviations

C1-INH: C1 inhibitor (C1 esterase inhibitor)
U: Unit
HAE: Hereditary angioedema
HAE-C1-INH: Hereditary angioedema due to C1 esterase inhibitor deficiency
pdC1-INH: Plasma-derived C1-INH concentrate

## References

[1] Bork K, Hardt J (2009). Hereditary angioedema: increased number of attacks after frequent treatments with C1 inhibitor concentrate. Am J Med.

[2] Bork K, Hardt J, Schicketanz KH, Ressel N (2003). Clinical studies of sudden upper airway obstruction in patients with hereditary angioedema due to C1 esterase inhibitor deficiency. Arch Intern Med.

[3] Bork K, Hardt J, Staubach-Renz P, Witzke G (2011). Risk of laryngeal edema and facial swellings after tooth extraction in patients with hereditary angioedema with and without prophylaxis with C1 inhibitor concentrate: a retrospective study. Oral Surg Oral Med Oral Pathol Oral Radiol Endod.

[4] Bork K, Siedlecki K, Bosch S (2000). Asphyxiation by laryngeal edema in patients with hereditary angioedema. Mayo Clin Proc.

[5] Bowen T (2011). Hereditary angioedema: beyond international consensus—circa December 2010—the Canadian Society of Allergy and Clinical Immunology Dr. David McCourtie Lecture. Allergy asthma. Clin Immunol.

[6] Bowen T, Cicardi M, Farkas H (2010). 2010 international consensus algorithm for the diagnosis, therapy and management of hereditary angioedema. Allergy Asthma Clin Immunol.

[7] Boyle RJ, Nikpour M, Tang MLK (2005). Hereditary angioedema in children: a management guideline. Pediatr Allergy Immunol.

[8] Cicardi M, Banerji A, Bracho F (2010). Icatibant, a new bradykinin-receptor antagonist, in hereditary angioedema. N Engl J Med.

[9] Cicardi M, Bork K, Caballero T (2012). Evidence-based recommendations for therapeutic management of angioedema owing to hereditary C1 inhibitor deficiency: consensus report of an International Working Group. Allergy.

[10] Cicardi M, Levy RJ, McNeil DL (2010). Ecallantide for the treatment of acute attacks in hereditary angioedema. N Engl J Med.

[11] Cinryze. Summary of product characteristics. Available at http://www.ema.europa.eu/docs/en_GB/document_library/EPAR_-_Product_Information/human/001207/WC500108895.pdf. Accessed 13 March 2012

[12] Craig TJ, Bewtra AK, Bahna SL (2011). C1 esterase inhibitor concentrate in 1085 hereditary angioedema attacks—final results of the I.M.P.A.C.T.2 study. Allergy.

[13] Craig TJ, Levy RJ, Wasserman RL (2009). Efficacy of human C1 esterase inhibitor concentrate compared with placebo in acute hereditary angioedema attack. J Allergy Clin Immunol.

[14] De Serres J, Gröner A, Lindner J (2003). Safety and efficacy of pasteurized C1 inhibitor concentrate (Berinert P) in hereditary angioedema: a review. Transfus Apher Sci.

[15] Farkas H, Harmat G, Gyeney L, Füst G, Varga L (1999). Danazol therapy for hereditary angio-oedema in children. Lancet.

[16] Farkas H, Jakab L, Temesszentandrási G (2007). Hereditary angioedema: a decade of human C1-inhibitor concentrate therapy. J Allergy Clin Immunol.

[17] Frank MM, Jiang H (2008). New therapies for angioedema: disease outlook changes dramatically. J Allergy Clin Immunol.

[18] Frank MM, Sergent JS, Kane MA, Alling DW (1972). Epsilon aminocaproic acid therapy of hereditary angioneurotic edema. A double-blind study. N Engl J Med.

[19] Garnock-Jones KP (2010). Ecallantide: in acute hereditary angioedema. Drugs.

[20] Gwynn CM (1974). Therapy in hereditary angioneurotic oedema. Arch Dis Child.

[21] Kalbitor. United States prescribing information. Available at http://www.accessdata.fda.gov/drugsatfda_docs/label/2009/125277lbl.pdf. Accessed 13 March 2012

[22] Kreuz W, Rusicke E, Martinez-Saguer I, Aygören-Pürsün E, Heller C, Klingebiel T (2012). Home therapy with intravenous human C1-inhibitor in children and adolescents with hereditary angioedema. Transfusion.

[23] Levy JH, Freiberger DJ, Roback J (2010). Hereditary angioedema: current and emerging treatment options. Anesth Analg.

[24] Levy RJ, Lumry WR, McNeil DL (2010). EDEMA4: a phase 3, double-blind study of subcutaneous ecallantide treatment for acute attacks of hereditary angioedema. Ann Allergy Asthma Immunol.

[25] Longhurst HJ, Farkas H, Craig TJ (2010). HAE international home therapy consensus document. Allergy Asthma Clin Immunol.

[26] Martinez-Saguer I, Rusicke E, Aygören-Pürsün E, von Hentig N, Klingebiel T, Kreuz W (2010). Pharmacokinetic analysis of human plasma-derived pasteurized C1-inhibitor concentrate in adults and children with hereditary angioedema: a prospective study. Transfusion.

[27] Maurer M, Magerl M (2010). Hereditary angioedema: an update on available therapeutic options. J Dtsch Dermatol Ges.

[28] Maurer M, Magerl M (2011). Long-term prophylaxis of hereditary angioedema with androgen derivatives: a critical appraisal and potential alternatives. J Dtsch Dermatol Ges.

[29] Sheffer AL, Austen KF, Rosen FS (1972). Tranexamic acid therapy in hereditary angioneurotic edema. N Engl J Med.

[30] Sheffer AL, Campion M, Levy RJ, Li HH, Horn PT, Pullman WE (2011). Ecallantide (DX-88) for acute hereditary angioedema attacks: integrated analysis of 2 double-blind, phase 3 studies. J Allergy Clin Immunol.

[31] Tallroth GA (2011). Long-term prophylaxis of hereditary angioedema with a pasteurized C1 inhibitor concentrate. Int Arch Allergy Immunol.

[32] Zanichelli A, Vachini R, Badini M, Penna V, Cicardi M (2011). Standard care impact on angioedema because of hereditary C1 inhibitor deficiency: a 21-month prospective study in a cohort of 103 patients. Allergy.

[33] Zuraw BL (2008). Hereditary angioedema. N Engl J Med.

[34] Zuraw BL, Busse PJ, White M (2010). Nanofiltered C1 inhibitor concentrate for treatment of hereditary angioedema. N Engl J Med.

[35] Zuraw BL, Cicardi M, Levy RJ (2010). Recombinant human C1-inhibitor for the treatment of acute angioedema attacks in patients with hereditary angioedema. J Allergy Clin Immunol.

